# Evaluation of artificial intelligence identified ipratropium bromide for the treatment of coronavirus disease 2019

**DOI:** 10.1038/s41598-025-27869-y

**Published:** 2026-02-19

**Authors:** Dong-Sub Jung, Seungwoo Cheon, Choon-Mee Kim, Jun-Won Seo, So Jung Sung, Da-Young Kim, Jae-Hak Lee, Wonwoo Kim, Jihoon Kang, Na-Ra Yun, Yi Rang Kim, Dong-Min Kim

**Affiliations:** 1 Bio-laboratory of ONCOCROSS Co., Ltd., 1301-ho, 127 Bubwon-ro, Songpa-gu, Seoul, Republic of Korea; 2 Artificial Intelligence Laboratory of ONCOCROSS Co., Ltd., #905 7 Beobwon-ro 11-gil, Songpa-gu, Seoul, Republic of Korea; 3 Division of Infectious Diseases, Departments of Internal Medicine, College of Medicine, Chosun University, Gwangju, Republic of Korea; 4 Department of Research Planning, ONCOCROSS Co., Ltd., #905 7 Beobwon-ro 11-gil, Songpa-gu, Seoul, Republic of Korea; 5 ONCOCROSS Co., Ltd., #905 7 Beobwon-ro 11-gil, Songpa-gu, Seoul, Republic of Korea

**Keywords:** Severe acute respiratory syndrome coronavirus 2 (SARS-CoV-2), Coronavirus disease 2019 (COVID-19), Ipratropium bromide, Remdesivir, Artificial intelligence, AI-aided drug development, Viral infection, Virtual drug screening, Drug development, Preclinical research

## Abstract

Ipratropium bromide (IB), identified through an artificial intelligence (AI)-aided drug screening platform, was evaluated for its safety and efficacy in treating severe coronavirus disease 2019 (COVID-19) using a hamster model in this study. The mortality rates in the vehicle, remdesivir-treated, IB-treated, and IB-remdesivir combination treatment groups were 70%, 15%, 5%, and 50%, respectively. The IB-treated group showed significantly lower blood D-dimer levels and fibrin degradation product when compared to that in the vehicle group, indicating prevention of thrombosis-related complications. Histological examination also revealed reduced lung inflammation in the IB and combination groups, compared to that in the vehicle and remdesivir groups, with fewer neutrophils in bronchoalveolar lavage fluid. Moreover, IB may prevent thrombosis-related complications caused by severe COVID-19.

**Supplementary Information:**

The online version contains supplementary material available at 10.1038/s41598-025-27869-y.

## Introduction

The coronavirus, initially discovered over 60 years ago, resurfaced in 2003 as SARS-CoV^1^, causing lethal respiratory illness. In November 2019, a new variant, SARS-CoV-2 emerged, leading to the global pandemic of coronavirus disease 2019 (COVID-19)^2^. Despite 2021 COVID-19 vaccine development^3^, the virus persists, leading to the emergence of new mutations^4^. Urgent action is needed to accelerate development of diverse therapeutic agents for effective COVID-19 treatment.

Human COVID-19 is characterized by respiratory symptoms^5^, pneumonia with increased inflammation^6^, fever^7^, chills, and potential secondary infections in brain or liver^8–10^. Additionally, blood clot-related complications, including deep vein thrombosis, pulmonary embolism, myocardial infarction, and ischemic stroke are observed^11,12^. Accurate evaluations of new COVID-19 drugs require an animal model replicating human symptoms. Existing models, however, regarding only respiratory symptoms and pneumonia^13–15^, are inadequate as human COVID-19 models leaving challenges in the development of COVID-19 vaccines and treatments. While mortality is rarely reported in immunocompetent hamsters^16^, Roborovski hamster strain SH101 post-infection of SARS-CoV-2 represented most clinical symptoms of COVID-19 such as snuffling, labored breathing, dyspnea, cough, hunched posture, progressive weight loss, ruffled fur, and high fever following shaking chills. Histological examinations also revealed initial right-predominated pneumonia as well as slight organ damages in the brain and liver, manifesting systemic COVID-19 cases. Considering the merit of a small animal as well as its clinical manifestations of SARS-CoV-2 infection in human, this hamster model seems to provide an ideal tool to investigate COVID-19^10^.

Ipratropium bromide (IB) is an anti-cholinergic agent that widens lung airways^17^, commonly used for asthma and chronic obstructive pulmonary disease (COPD)^18^. This study evaluates the potential repositioning of IB in COVID-19 treatment, as suggested by the commercial artificial intelligence (AI) platform RAPTOR AI.

## Materials and methods

### Ethics statement

This study was approved by the Institutional Review Board (Project title: A Randomized, Open-Label, Controlled Clinical Trial of Remdecivir Plus Ipratropium Bromide Versus Remdecivir Alone in Patients With Severe COVID-19, approval number: 2021-01-011-001). According to the principles of the Declaration of Helsinki, signed written consent was obtained from each participant or their legal guardian for the use of data and samples for scientific purposes.

All animal experiments were approved by the Institutional Animal Care and Use Committee (IACUC) of Jeonbuk National University (approval title: “Evaluation of the Efficacy of Ipratropium Bromide Against SARS-CoV-2”; approval No. JBNU 2021 − 0184; Ethics Committee approval No. NON 2022 102) and were conducted in accordance with institutional regulations and the ARRIVE guidelines and checklist.

### Preparation of total RNA of COVID-19 patients’ blood samples

Eight COVID-19 patients (≥ 19 years old) in South Korea were enrolled from February 2020 to January 2021. The diagnosis of COVID-19 in the enrolled patients was confirmed using molecular methods. Specifically, we utilized our in-house-designed reverse transcription polymerase chain reaction (RT-PCR) targeting the N gene, along with a commercial kit (Kogene Biotech, Seoul, South Korea) targeting the E and RdRp genes, following the manufacturer’s protocol. A positive COVID-19 diagnosis was determined if more than two genes were detected at a Ct value < 38 or if the SARS-CoV-2 culture yielded a positive result^19^. Regarding lung imaging, all severe cases exhibited pneumonic changes in chest imaging, whereas mild cases did not show significant abnormalities. Among them, five had mild symptoms, and three had severe symptoms on admission. Disease severity was classified based on the World Health Organization (WHO) COVID-19 severity criteria (https://bestpractice.bmj.com/topics/en-gb/3000201/criteria):

- Mild illness: Symptomatic patients meeting the case definition for COVID-19 without evidence of hypoxia or pneumonia.
- Severe disease (adolescent or adult): Clinical signs of pneumonia (i.e., fever, cough, dyspnea, fast breathing) plus one of the following:
  - Respiratory rate > 30 breaths/minute.
  - Severe respiratory distress.
  - SpO₂ <90% on room air.

All severe cases in our study met at least one of these criteria, while mild cases did not exhibit hypoxia or pneumonia. We have clarified this classification in the revised manuscript.Blood samples were collected from each patient at diagnosis and convalescence. Human peripheral blood mononuclear cells (PBMCs) were isolated using Ficoll Paque Plus (Sigma-Aldrich, Burlington, MA, USA) following density gradient centrifugation^20^. Total RNAs were isolated from PBMCs using the TRI Reagent (Molecular Research Center, Inc., Cincinnati, OH, USA).

**Table:** 

Age/sex	Disease severity	Comorbidities	Pneumonia on imaging	SpO_2_ in admission	Oxygen therapy	Steroid use
35/F	Mild	None	No	98%	No	No
22/M	Mild	None	No	98%	No	No
46/M	Mild	HTN, Hyperlipidemia	No	97%	No	No
30/M	Mild	HTN	No	97%	No	No
30/M	Mild	None	No	98%	No	No
79/F	Severe	HTN, DM, Guillain-Barre syndrome	Yes	85%	Yes	Yes
61/F	Severe	None	Yes	89%	Yes	No
75/F	Severe	HTN, old CVA, Lt.hemiparesis	Yes	88%	Yes	No

### Data sources for drug screening analysis

The mRNA levels of the collected sixteen blood samples were measured by QuantSeq 3′ mRNA sequencing conducted by e-Biogen (Seoul, Korea)^21^. The raw FASTQ RNA sequencing data were processed using conventional NGS pipeline tools, including Trimmomatic (version 0.39-1) and Salmon (version 0.7.2), for trimming and alignment processes, respectively, producing quantified transcriptome data in transcripts per million (TPA). Transcriptome datasets for 23,277 chemicals were collected from large-scale databases, including the Connectivity Map and Library of Integrated Network-based Cellular Signatures L1000.

### RAPTOR AI

**Fig. 1:**
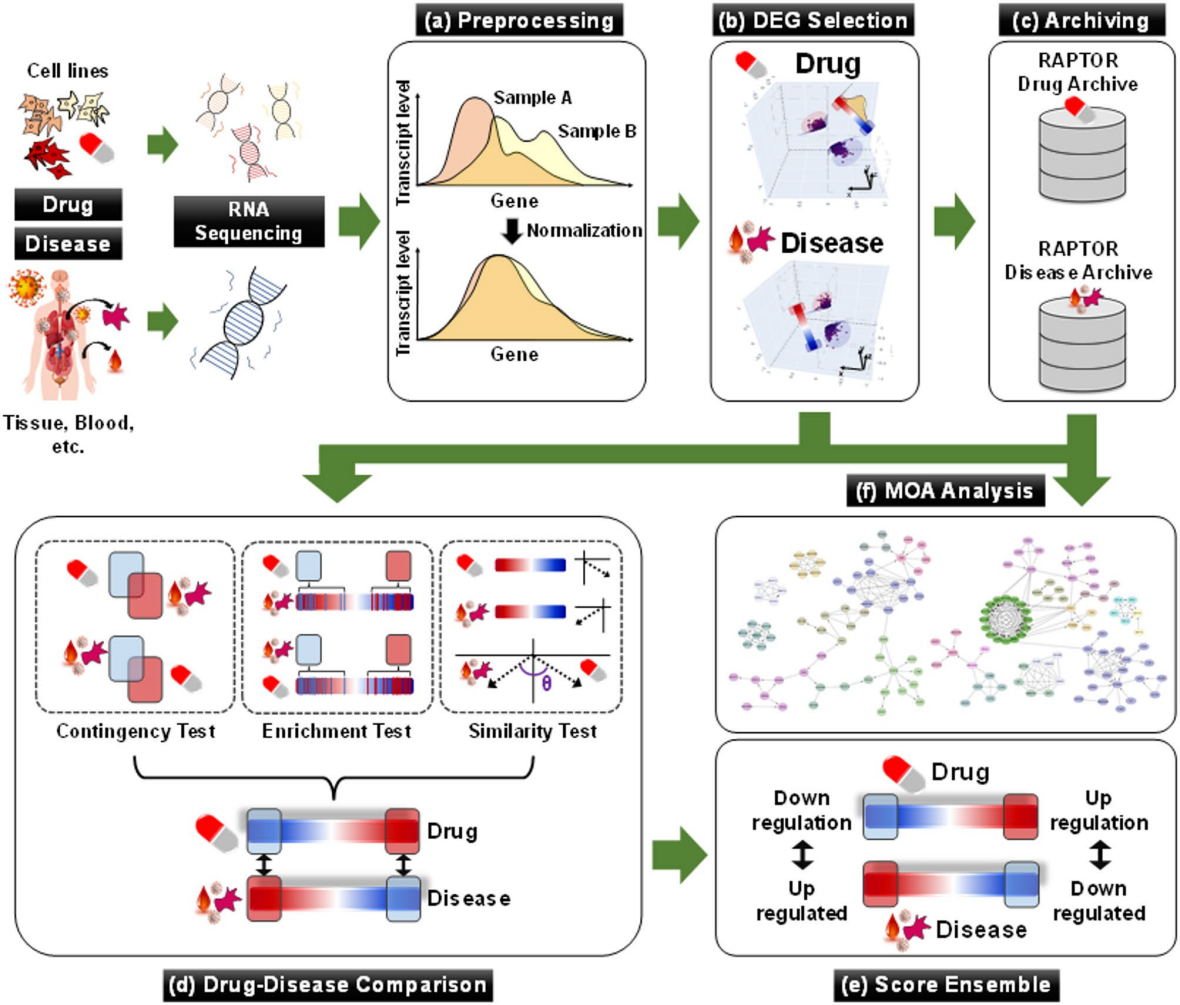
Overall analytic process of RAPTOR AI. (**a**) RNA datasets are standardized and normalized through preprocessing before use in the drug-screening process. (**b**) Statistically significant DEGs are identified between normal and disease samples, as well as between drug-treated samples and their negative controls. (**c** and** f**) All processed data is stored in proprietary databases for future studies, including MOA analysis. (**d**) The Drug-disease comparison analyzes the complementary correlation between drug-derived and disease-derived DEGs using three statistical tests: the contingency test (CT), the enrichment test (ET), and the similarity test (ST). (**e**) The final chemical score is determined in the score ensemble process.

RAPTOR AI (ONCOCROSS Co., Ltd., Seoul, Korea) is a commercial AI drug-screening platform with four analytical processes: collection and preprocessing of transcriptome datasets, differently expressed gene (DEG) selection, drug-disease comparison, and score ensemble (Fig. 1)^22^. The analysis utilized both drug- and disease-derived RNA expression datasets to identify ideal drug candidates (see details in Supplementary Methods).

### Signaling pathways and mode of action analysis

A web-based pathway analysis tool, the Consensus Pathway Analysis (CPA), was utilized for the pathway analysis^23^. Detailed analysis of mode of actions (MOAs) among DEGs (Table S1) identified by RAPTOR AI within pathways was conducted using the Kyoto Encyclopedia of Genes and Genomes pathway database, following the standard analytical protocol^24^. *P*-values < 0.05 were considered statistically significant.

### Compounds

IB and remdesivir were purchased from Selleck Chemicals, LLC (Houston, TX, USA). IB was administered to the whole body using a nebulizer, and remdesivir was dissolved in dimethyl sulfoxide (DMSO; Sigma-Aldrich, St. Louis, MO, USA) and subsequently diluted with saline to the final concentration of the target dose.

### Cell and virus

Vero cells were purchased from the Korean Cell Line BANK. The SARS-CoV-2 strain was obtained from the National Culture Collection for Pathogens of the Korea Disease Control and Prevention Agency. The virus was proliferated in Vero cells at 37 °C, 5% CO_2_, and subsequently harvested for our experiments. Genome details of the virus strain are available at GISAID (identifier: BetaCoV/Wuhan/IVDC-HB-01/2020| EPI_ISL_402119) and the China Microbiology Data Center (accession number NMDC10013001 and genome accession number MDC60013002-01).

### Cell culture

Vero cells were cultured in Dulbecco’s modified Eagle’s medium (DMEM; Gibco, Waltham, MA, USA), supplemented with 10% FBS, 1% penicillin-streptomycin, at 37 °C, 5% CO_2_. A549-ACE2-TMPRSS2 and THP-1 cells were co-cultured in Roswell Park Memorial Institute 1640 medium, supplemented with 10% FBS, 1% penicillin-streptomycin, at 37 °C, 5% CO_2_.

### Cell viability assay

Compound cytotoxicity was assessed using WST-1 cell proliferation reagent (Roche, Basel, Switzerland). Vero cells (2 × 10^4^ cells/well) were seeded in a 96-well clear flat-bottom TC-treated microplate with DMEM and incubated at 37 °C, 5% CO_2_. The next day, cells were washed once with phosphate-buffered saline (PBS) and serial two-fold dilutions of the cell suspension were inoculated into fresh medium. At 48 h post-infection, 10 µL of WST-1 was added to each well, followed by 2 h of incubation at 37 °C and 5% CO_2_. Cell viability was determined by measuring absorbance using a microplate reader.

### Virus infection and cell harvest

Vero cells were seeded in 24-well plates until attachment. After a cell wash with fresh DMEM, the cells were inoculated with 0.01 multiplicity of infection (MOI) SARS-CoV-2, or with DMEM for negative control, then incubated at 33 °C for 1 h for virus adsorption. After drug treatment tests, the cell pellets were collected at specified time points and stored at -80 °C for RNA extraction.

### Animal study

Inbred SH101 hamsters of the same genetic background were utilized in assessment of IB efficacy in treating COVID-19. Four-month-old hamsters were purchased from Alpha Biochemicals (Torrance, CA, USA) and were randomly divided into groups of no more than five hamsters per cage in the Animal Biosafety Level 3 Animal Breeding Room. All animal maintenance and experimental procedures were performed according to the regulations and procedures of the facility.

### Evaluation of therapeutic efficacy

The COVID-19 animal model in SH101 hamsters was established by intranasal injection of 50 µL SARS-CoV-2 aqueous solution (10^5^ TCID_50_). Treatment details for each group are in Table S2. The healthy control received 50 µL DMEM, while infection control group was given the viral solution. Remdesivir or IB administered in a weight-dependent manner. According to the scheduled experimental timeline, all hamsters euthanized under anesthesia with isoflurane followed by cervical dislocation.

A remdesivir solution was prepared by dissolving the drug in DMSO, filtering through a 0.45-µm syringe filter, and diluting with saline to a 15 mg/kg concentration. The prepared remdesivir solution was intravenously administered to appropriate groups from 1 to 8 day post-infection (dpi).

For IB treatment, a 500 µg/2 mL stock solution was diluted to 62.5 µg/kg with saline. Hamsters in the IB group received IB in nasal cavity via nebulization using a peristaltic pump (Fig. S1).

**Table:** 

Group		Sacrifice			Total
		2 dpi	5 dpi	8 dpi	
Normal animal	Control	5	5	5	15
Disease animal	Vehicle	5	5	5	15
	Remdesivir	5	5	5	15
	Ipratropium bromide	5	5	5	15

### Hamster clinical observation

A thermal imaging camera (FLIR, Wilsonville, OR, USA) was utilized to capture the thermal images of the whole bodies of hamsters from 0 to 8 dpi. The DirA (FLIR Tools) was then employed to identify the highest temperature point.

Body weight was measured at 0–8 dpi using a scale. The rate of weight change (%) was calculated as [current weight/0 dpi weight × 100%].

Student’s t-test was conducted using the mean and standard deviation values to identify significance on differences between groups.

### Blood analysis

At 2, 5, and 8 dpi, whole blood was collected from each hamster group by cardiac puncture. Whole blood was centrifuged at 2,500 g for 10 min to separate the serum. The serum was immediately stored at -80 °C.

The D-dimer and fibrin degradation products (FDP) levels were quantified by enzyme-linked immunosorbent assay (ELISA) using ELISA kits (D-dimer: Cat. No. MBS012417, FDP: Cat. No. MBS005821, MyBioSource, San Diego, CA, USA). The samples were diluted 10-fold following the manufacturer’s instructions. Subsequently, a 50 µL of the diluted sample was dispensed into a well on a D-dimer or FDP antibody-coated micro-ELISA plate and incubated at 37 °C for 60 min. Then, the chromogen solution was added and the mixture reacted for 15 min. Immediately after terminating the reaction by adding the stop solution, the OD value was measured at 450 nm using an ELISA reader (iMark microplate reader, Cat No. #1681130; Biorad, Hercules, CA, USA). The OD450 values were normalized based on a blank OD450. The protein concentrations were calculated from the OD450 values using standard curves generated using six standard samples for D-dimer (31.2 ng/mL to 1,000 ng/mL) or FDP (0.625 pg/mL to 10 pg/mL) respectively.

The interleukin (IL)-6and tumor necrosis factor (TNF)-⍺ levels were quantified using ELISA kits (IL-6: Cat. No. MBS7606648, TNF-⍺: Cat. No. MBS7606475). The sample loading procedures were identical to those described above, except that a 100 µL sample was used instead of 50 µL, and the incubation time on the antibody-coated plate was extended to 90 min instead of 60 min. Then, a biotin-labeled antibody solution was added and allowed to react, followed by washing with a wash buffer. Horseradish peroxidase-streptavidin conjugate was added, reacted, and then washed. After adding the chromogenic substrate, the mixture reacted according to the manufacturer’s instructions, until the stop solution was added to stop the reaction. Calculation IL-6 and TNF-⍺ levels were calculated using the same procedure as described above, utilizing the OD450 values and standard curves of IL-6 (7.8 pg/mL to 500 pg/mL) and TNF-⍺ (3.125 pg/mL to 200 pg/mL).

### Autopsy and histopathology

SH101 hamsters at 2, 5, and 8 dpi were euthanized and lungs were isolated by laparotomy. Whole-lung images were captured using a digital camera. Immediately, the right lung lobe was fixed using 10% neutral formalin. After washing with water followed by dehydration with ethanol, the tissue was made transparent using xylene and embedded in paraffin via a penetration process. Sections of 5 μm were cut, attached to glass slides. After deparaffinization and dehydration, lung tissue samples were subjected to H&E and IHC staining following general procedures (Supplementary Methods) and observed under optical microscopy.

### Quantification of the SARS-CoV-2 virus in lung tissues of SH101 hamsters

At 2, 5, and 8 dpi, SH101 hamsters were euthanized and dissected to isolate the lungs. Left lung lobe was homogenized with a tissue homogenizer and centrifuged at 3,000 rpm for 10 min at 4 °C. The supernatant was carefully collected, and total RNA was extracted using the RNeasy Mini Kit (QIAGEN, Hilden, Germany) according to the manufacturer’s manuals. The isolated total RNA was used as template in the subsequent RT-qPCR (Supplementary Methods).

Real-time PCR standard curves generated using serial dilution of recombinant plasmids. The log10 transformations of the equivalent nucleotide copy number per mL of the samples were used to express the viral titer. Statistical significance between groups was evaluated by a one-way ANOVA multiple comparison test at *P <* 0.05 using the mean ± standard deviation.

### Bronchoalveolar lavage fluid (BALF) test

At 2, 5, or 8 dpi, neck of pre-euthanized hamster was dissected and a catheter was inserted into the bronchus and secured with a suture. Subsequently, 1 mL PBS was circulated three times to obtain BALF. Cytospin centrifugation was used to separate the cells from the BALF solution before drying the slides. Giemsa staining solution (Cat No. GS-500, Sigma-Aldrich) was used to stain the dried slides. The stained slides were covered with mounting solution and cover glass, and infiltrated neutrophils were examined under an optical microscope.

## Results

### Transcriptome-based drug screening

**Fig. 2:**
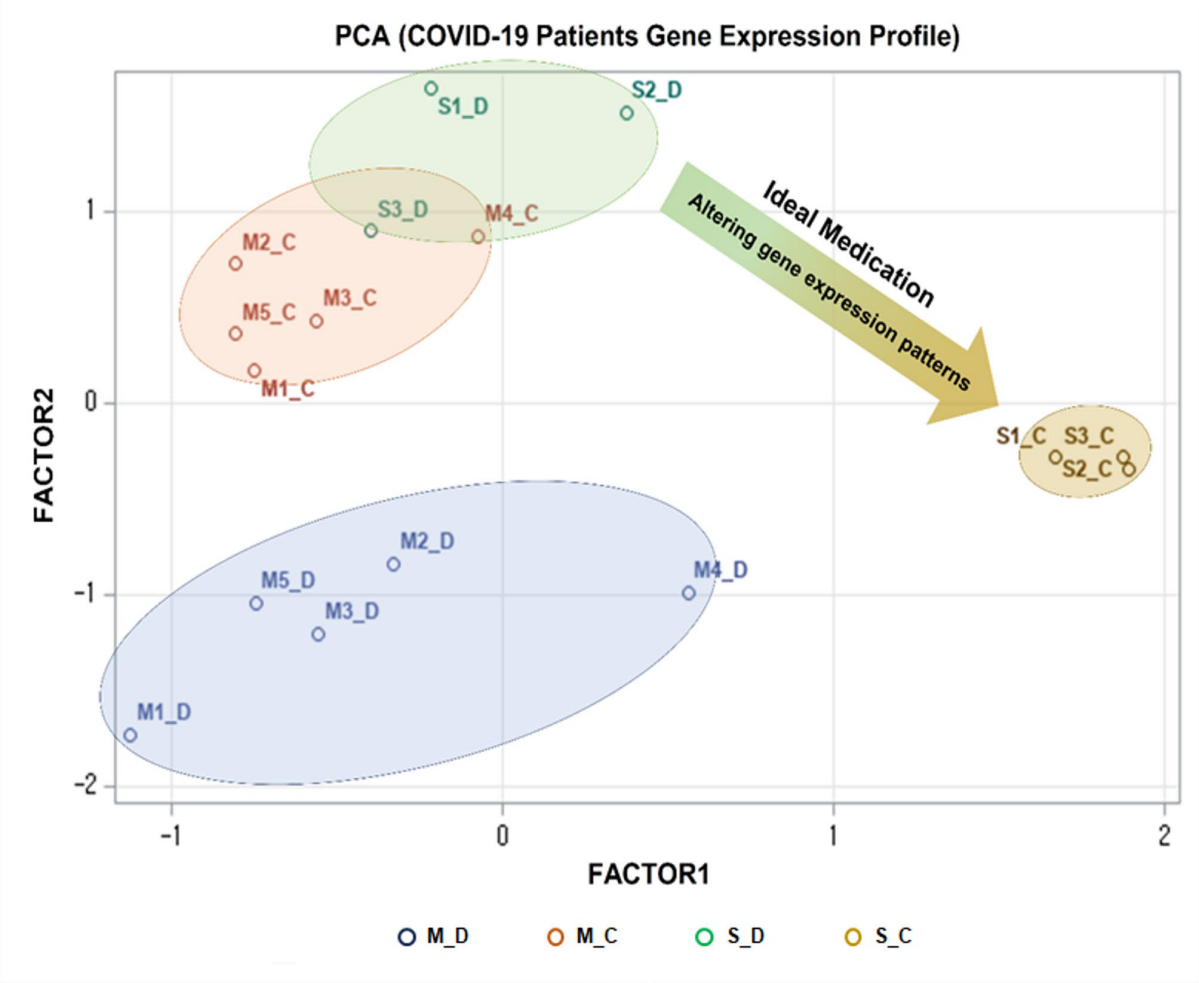
Principal component analysis (PCA) results between RNA expression patterns of patients infected with COVID-19. The label M and S denoted mild or severe symptoms, respectively. Specifically, five patients with mild symptoms were designated as M1 to M5, while three patients with severe symptoms were labeled as S1 to S3. The labels D and C, appended after each patient label (e.g., M1_D and M1_C), signified the patients at diagnosis and the patients at convalescence, respectively.

In Principal component analysis (PCA) the transcript expression patterns of patients’ blood samples were distinguishable into four major groups based on the degree of infection and the disease progression period (Fig. 2). The interesting point is that the difference in gene expression patterns among patients with severe symptoms was significantly exclusive, depending on the patients’ disease progression status. In this sense, the datasets of severe patients were used in further analysis.

RAPTOR AI algorithm pursued chemicals capable of regulating imbalanced transcript expression patterns in severe patients at diagnosis, resembling the convalescence, yielding a list of putative candidates, with IB being a top candidate for COVID-19 (Table 1). A commercial COVID-19 drug, remdesivir, was ranked third among 23,377 drugs, fortifying the feasibility of the drug screening method.

**Table 1:** The rankings of putative drug candidates for COVID-19 resulted from an artificial intelligence-aided drug-screening method.

Rank	Chemical	Rank	Chemical	Rank	Chemical
1	Ipratropium bromide	11	BRD-A30083233	21	BRD-K07237640
2	BRD-K38467418	12	BRD-K68211814	22	BRD-K08447949
3	Remdesivir	13	BRD-K08540981	23	BAS-09104376
4	BRD-K74126469	14	Caffeine	24	BRD-K11062991
5	CTB	15	BRD-K63375110	25	KU-C103875
6	BRD-K00442147	16	GSK-429,286 A	26	Gallopamil
7	Skimmianine	17	BRD-K24932095	27	BRD-K81314178
8	Actarit	18	Pantoprazole	28	BRD-K27835062
9	BRD-K83691318	19	BRD-A16581344	29	Amfepramone
10	BRD-K15638296	20	PF-04620110	30	Enalapril

### Signaling pathways and IB-related MOAs regarding COVID-19 treatment

IB was previously identified as an anti-cholinergic agent blocking acetylcholine-mediated inflammatory pathways^25,26^. Therefore, the key MOA of IB in COVID-19 treatment was suspected to be related to its anti-inflammatory effect.

A list of pathways was identified through CPA using DEGs of severe COVID-19 patients, and, as hypothesized, a few pathways were found to be related to immune and anti-inflammatory responses (Table S3).

Based on the anticipated action of IB for COVID-19 treatment, enrichment analysis of the DEGs in eight inflammatory response-related pathways suggested a hypothetical in-depth MOA of IB in curing COVID-19.

### Evaluation of IB for body temperature recovery

**Fig. 3:**
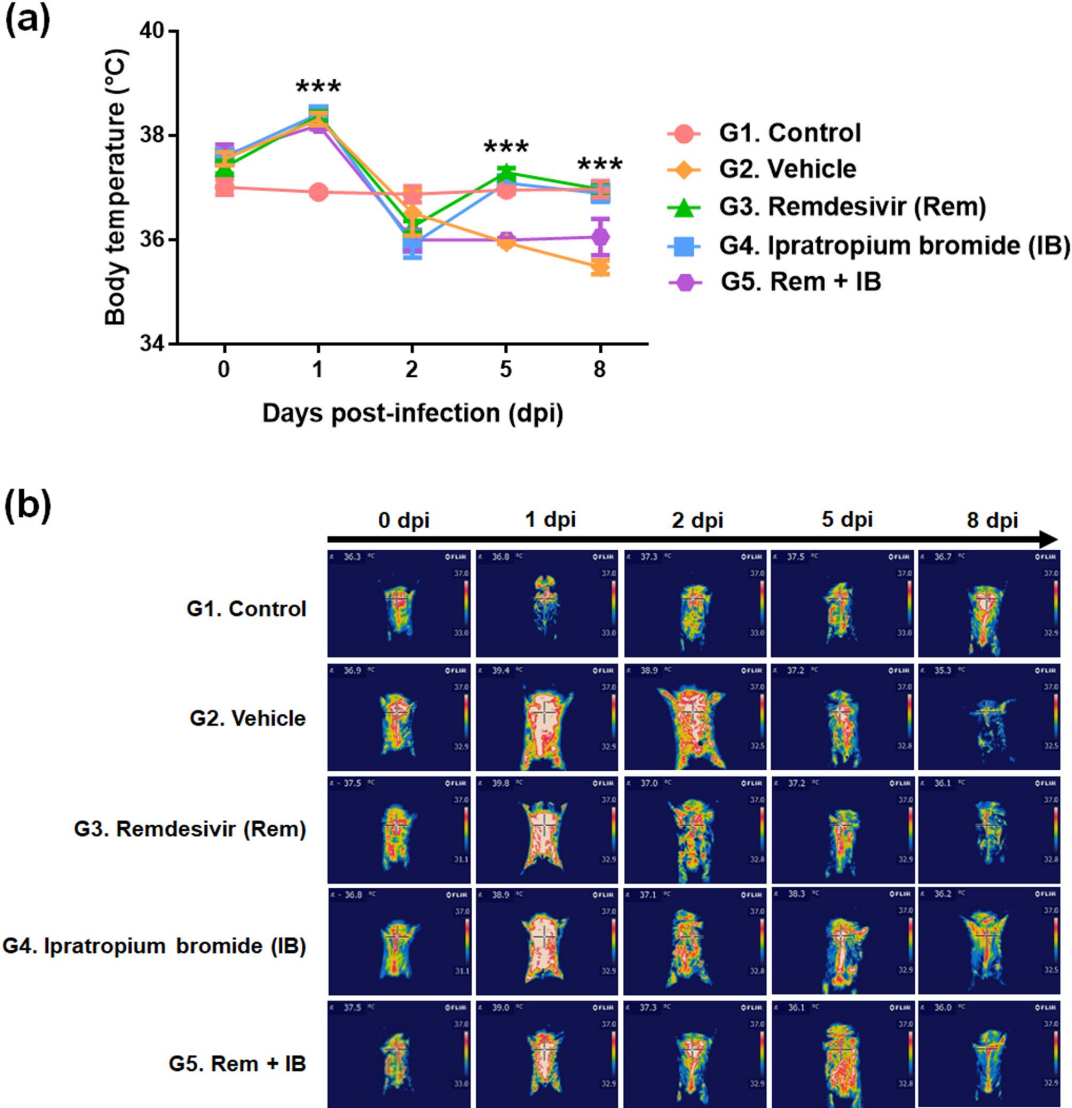
Effect of ipratropium bromide (IB) on changes in body temperature in SH101 hamsters infected with the SARS-CoV-2 virus. (**a**) The SH101 hamster’s body temperature was measured by measuring the chest surface temperature closest to the lungs. Changes in body temperature were measured for 9 days (0–8 days post-infection). Body temperature was measured by selecting the highest temperature point in the thermal image. Each data was expressed as the mean ± standard deviation (*n* = 15). (**b**) Representative infrared thermal images for each experimental group are displayed for body temperature measurement. Statistical significance compared to the infection control group was indicated in the graph as *P < 0.05, **P < 0.01, and ***P < 0.001.

Fever is a common in infectious diseases, including COVID-19, caused by the innate immune response. Severe infections can result in hypothermia due to the deterioration of physiological functions after the early onset of high fever, leading to death^27^. In our study, the control group hamsters infected with SARS-CoV-2 exhibited a high fever at 1 dpi, followed by rapid hypothermia at 2 dpi, with the body temperature continuing to drop until death, representing a severe COVID-19 pattern. In contrast, healthy hamsters maintained a normal body temperature (36.5 °C) throughout the experiment. The drug treated groups (G3, G4, and G5) exhibited hypothermia from 2 dpi, resembling vehicle group, but body temperature began to recover from 5 dpi in remdesivir (G3) and IB (G4) groups (Fig. 3).

### Safety IB as drug

**Fig. 4:**
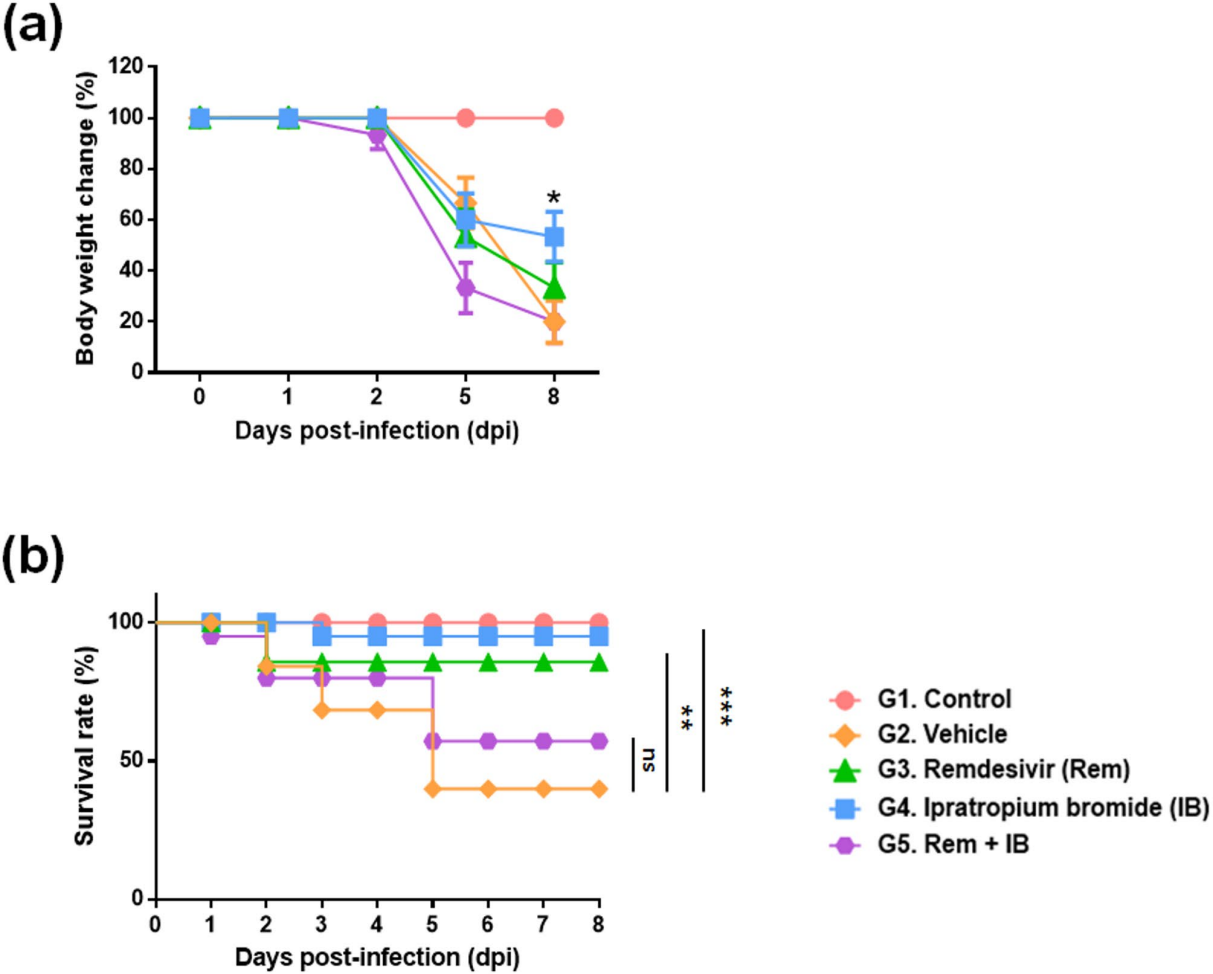
Effect of ipratropium bromide (IB) on changes in body weight and survival rate in SH101 hamsters infected with SARS-CoV-2. (**a**) Weight change was measured for 9 days (0–8 days post-infection). (**b**) The survival rate was measured for 9 days (0–8 dpi). Each data was expressed as the mean ± standard deviation (*n* = 15). The final mortality rate in the IB group (5%) improved more than that in the vehicle group (70%). Statistical significance compared to the infection control group was indicated in the graph as *P < 0.05, **P < 0.01, and ***P < 0.001.

Hamsters infected with SARS-CoV-2 exhibited a weight loss of 34 ± 9% at 5 dpi compared to their initial weight, which continued to decrease until death. At 8 dpi, the remdesivir group demonstrated a weight loss of 37.7 ± 1.2%, while the IB group and the combination group exhibited a weight loss of 11.6 ± 3% and 39.4 ± 2.1%, respectively (Fig. 4a). The mortality rates in the remdesivir (15%), IB (5%), and combination (50%) groups were lower compared to the vehicle group (70%) (Fig. 4b).

### Effect of IB on thrombosis and pro-inflammatory cytokine expression

**Fig. 5:**
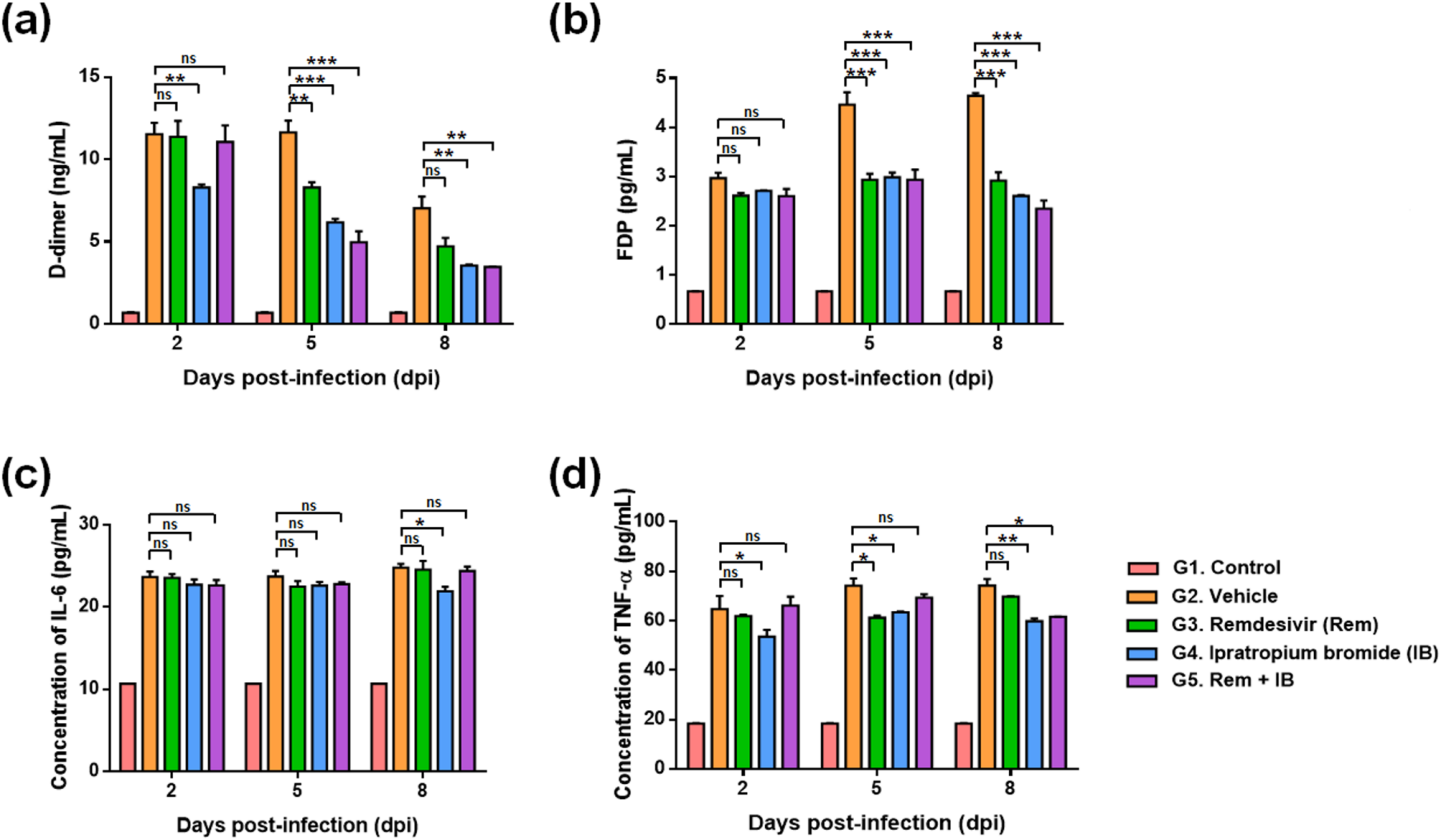
Changes in blood clotting factor content and inflammatory cytokines in SARS-CoV-2 virus-infected SH101 hamsters treated with ipratropium bromide (IB). The hypercoagulable state of the SH101 hamsters infected with SARS-CoV-2 was measured. The contents of (**a**) D-dimer, (**b**) fibrin degradation product, (**c**) interleukin-6, and (**d**) tumor necrosis factor-α in the serum of the SH101 hamster were obtained at 2, 5, and 8 dpi. Statistical significance compared to the infection control group was indicated in the graph as *P < 0.05, **P < 0.01, and ***P < 0.001.

One of the main characteristics of severe COVID-19 is the formation of blood clots in blood vessels which is related to complications such as myocardial infarction and ischemic stroke^28,29^. Especially, D-dimer and FDP are the major indicators used to evaluate blood clot formation. In this study, we investigated the effect of IB on blood clot formation in hamsters with COVID-19. The serum D-dimer levels were significantly lower in the IB group than that in the vehicle group (2 dpi: 20%, *P <* 0.01; 5 dpi: 40%; and 8 dpi: 75%, *P* < 0.001). D-dimer levels in the combination group significantly decreased at 5 (*P* < 0.01) and 8 dpi (*P* < 0.001). However, no significant difference was observed between the remdesivir and vehicle groups at 8 dpi (Fig. 5a). The blood FDP content in the treatment groups was significantly lower than that in the vehicle group at 2 (10%), 5 (33%), and 8 dpi (44%) (Fig. 5b). These results indicate the potential of IB in preventing thrombosis-related complications associated with COVID-19. The blood thrombogenicity factor in the IB group was lower than that in the remdesivir group.

Blood levels of pro-inflammatory and anti-inflammatory cytokines, such as IL-6 and TNF-⍺, is an important indicator for inflammation induced by pathogen invasion and subsequent infection. In this study, IB group showed a reduction in the IL-6 levels by 4.3% (2 dpi), 8% (*P* < 0.01, 5 dpi), and 20% (*P* < 0.01, 8 dpi) compared to the corresponding values in the vehicle group (Fig. 5c). Similarly, the TNF-⍺ levels were lowered by 18% (*P* < 0.01, 2 dpi), 14% (*P* < 0.05, 5 dpi), and 20% (*P* < 0.05, 8 dpi) in the IB group relative to the vehicle group (Fig. 5d). Among the drug-treated groups, no statistically significant difference in the pro-inflammatory cytokine levels were observed.

### Evaluation of IB on anti-inflammation effect in lungs

**Fig. 6:**
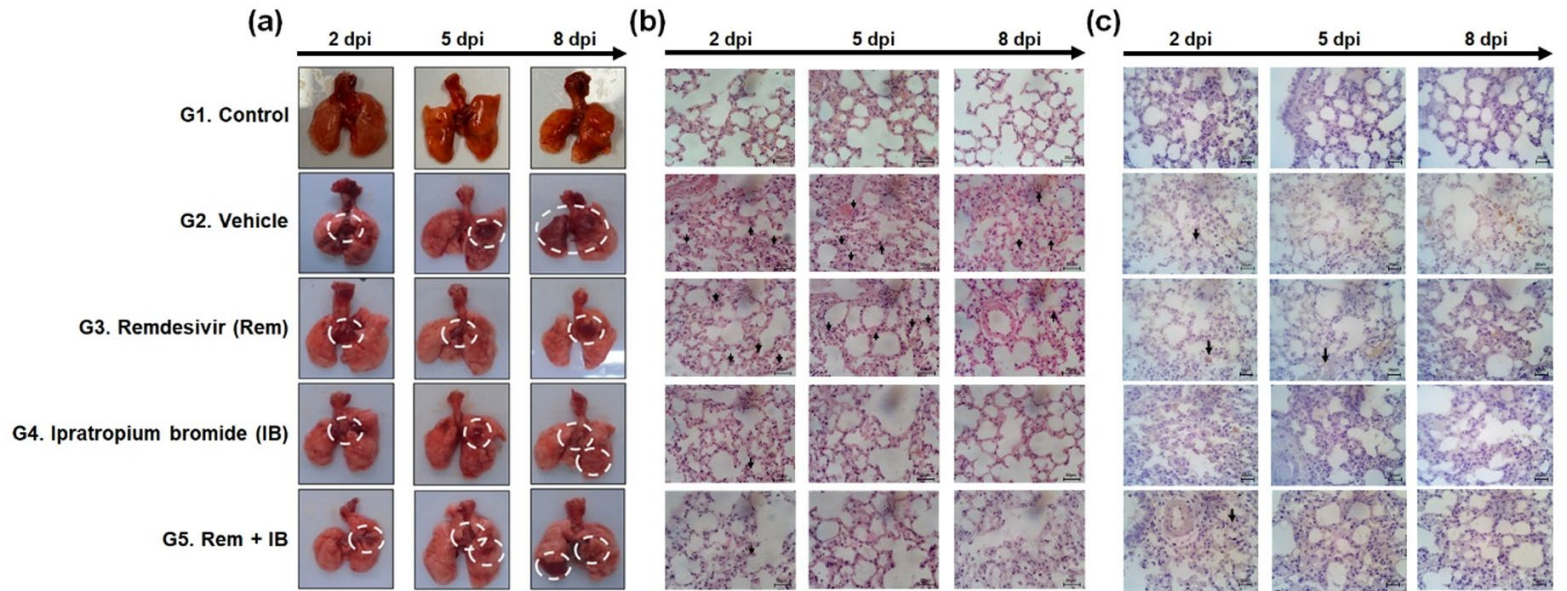
Macroscopic (**a**) and microscopic (**b**,**c**) observations in infected SH101 hamsters at 2, 5, and 8 days post-infection. (**a**) Gross finding of lungs from necropsied SH101 hamsters. (**b**) Hematoxylin and eosin-stained lung images of SH101 hamsters. Multifocal interstitial pneumonia infiltrated with inflammatory cells (black arrow) is indicated. (**c**) Immunohistochemistry-stained lung images of SH101 hamsters. An increase in the brown area of the lung tissue was observed with the degree of viral infection (black arrow).

Hamster lungs were histologically examined to assess the degree of inflammation (Fig. 6).

The vehicle group displayed worsening inflammatory lesions over time, while the drug treated groups showed less overall lung inflammation. Specifically, the IB group showed lowest lung inflammation among the drug treatment groups.

A significant increase in inflammatory cells of lung tissue was observed in vehicle group, and the number of inflammatory cells increased as the degree of infection worsened over time (Fig. 6b). The inflammation degree was lower in the remdesivir group than in the vehicle group, which was sufficient to demonstrate a clear reduction in inflammation. The lungs of the IB and combination groups did not differ significantly from those of the control group, indicating the absence of lung inflammation.

The extent of brown staining suggested extensive pulmonary inflammation in the vehicle group owing to SARS-CoV-2 infection (Fig. 6c). At 2 dpi, brown staining was observed in all experimental groups, with less brown staining in the lungs of the remdesivir group than in the vehicle group but more than IB. In the IB and combination-treatment groups, the brown-stained area gradually decreased over time, with a significant decrease observed at 8 dpi. These results are consistent with those of the other tests in this study, showing that IB is more effective than remdesivir in treating COVID-19, with less inflammation occurring in the lungs.

**Fig. 7:**
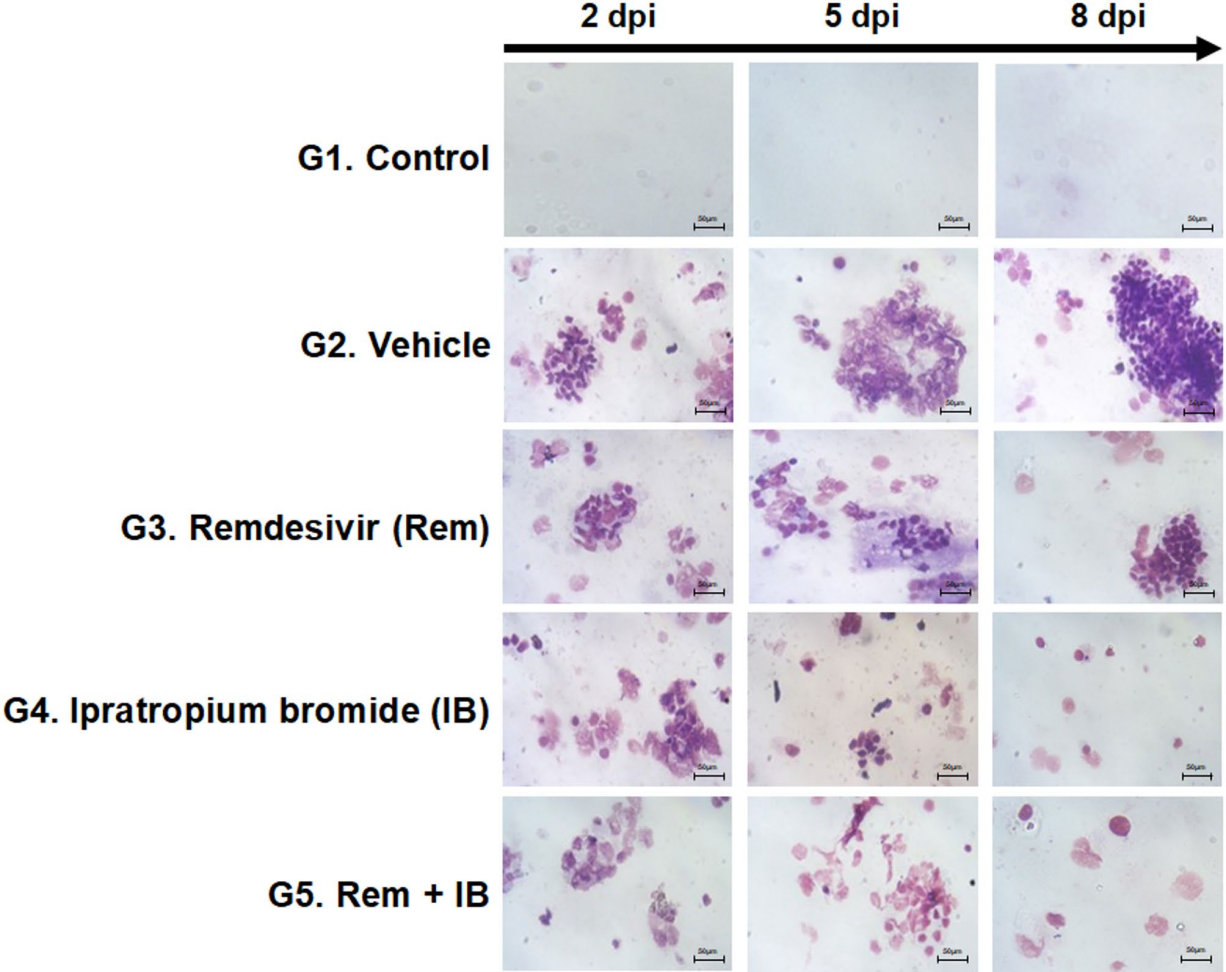
Effect of ipratropium bromide (IB) on reducing inflammation through Giemsa staining of BALF. When Giemsa-stained neutrophils were obtained at 2, 5, and 8 days post-infection, it was observed that the lung cells were stained purple as the number of neutrophils increased.

The vehicle group exhibited a notable rise in neutrophil inflammatory cells in lungs, corresponding to an increase in infection severity over time (Fig. 7). Overall, the neutrophil number in the alveolar lavage fluid of the IB and combination groups was significantly lower than that in the remdesivir group, suggesting that IB was more effective at inhibiting inflammation than remdesivir.

## Discussion

IB was the first inhalable muscarinic inhibitor for asthma or COPD patients^30^. A previous report demonstrated its use as a bronchodilator to alleviate COVID-19 symptoms^31^. Other studies have shown IB’s inhibition of SARS-CoV-2 binding in an in vitro experiment^32^. However, the efficacy of IB inhalation in inhalant form has never been evaluated in a COVID-19 animal model.

In the human lung, stimulation of M2 receptor induces fibroblasts proliferation. Acetylcholine enhances cell proliferation in cells isolated from COPD patients compared to healthy controls, involving extracellular signal-regulated kinase (ERK) 1/2 and nuclear factor kappa B (NF- κB) activation in lung fibroblasts. IB induces bronchodilation, tachycardia, and inhibits salivary secretion. Unlike atropine, IB lacks an appreciable effect on the central nervous system, is poorly absorbed by the lungs or gastrointestinal tract, and does not inhibit mucociliary clearance^33^.

Recent studies have identified virus-mediated phosphorylation or activation of ERK1/2, c-Jun N-terminal kinase, p38, phosphoinositide 3-kinase (PI3K)-Akt, and NF-κB signaling pathways that may contributed to lung injuries in COVID-19^34,35^. The MOA analysis conducted in this study aligns with and support the earlier findings^25,26,34^. Genes among the DEGs enriched in the top eight pathways (Table S3) were closely linked to the mitogen-activated protein kinase (MAPK) and PI3K-Akt signaling pathways, leading to alterations in the platelet transcriptome and proteome, causing hyperreactivity^34,36^. In summary, the modulation of MAPK or PI3K-Akt signaling is the key hypothetical mechanism of IB in address COVID-19. While this study provided a comprehensive hypothesis of IB’s MOA for COVID-19 treatment, focusing on inflammatory aspects, other top-ranked pathways may offer valuable insights for future research on IB in various infectious diseases.

Inflammation-induced mucus and narrowing of the pulmonary airways, common manifestations with COVID-19, hinders the immune interaction against pathogens^37,38^. Combining IB’s original medication purpose, our findings demonstrated an excellent therapeutic effect of IB administration as a respiratory drug in the treatment of COVID-19.

The lower mortality rate in the IB-treated group compared to the remdesivir-treated group demonstrated the reliable safety of IB (Fig. 4). With IB treatment, a significant reduction in lung inflammation was visually observed in biopsy and alveolar lavage fluid (Fig. 6, and Fig. 7). Molecular-level analysis further supported the effective anti-inflammation capacity of IB, displaying a clear reduction in thrombogenic and inflammatory factors levels with IB treatment (Fig. 5). Furthermore, it is expected that IB can also prevent thrombosis-related complications, such as myocardial infarction and ischemic stroke accompanying COVID-19^28,29^. In conclusion, the findings in study provides evidence that IB can serve an alternative COVID-19 drug which is safe and effective.

Moreover, the observed synergistic effects in combinatorial use of IB with remdesivir suggest the potential for real-world application (Fig. 5, Fig. S2, and Fig. S3). Nevertheless, further research on drug-to-drug interactions between IB and remdesivir seems necessary to address the toxicity issues exclusively observed in the in vivo study (Fig. 4) for actual clinical applications.

Finding a putative drug candidate is considered a “bottleneck” in the drug development process because of the tremendous amounts of spent resources, including time, effort, and money. This area has provided powerful AI tools that encourage the utilization of such platforms in pharmaceutical research, such as if RAPTOR AI screens IB as a potential treatment candidate for patients with COVID-19.

## Supplementary Information

Below is the link to the electronic supplementary material.

Supplementary Material 1

## Data Availability

The public databases used in the study: Connectivity Map (CMap): [https://www.broadinstitute.org/connectivity-map-cmap](https:/www.broadinstitute.org/connectivity-map-cmap) /; Library of Integrated Network-based Cellular Signatures (LINCS): [https://lincsproject.org/](https:/lincsproject.org) .
