# Supplementary material for: Evaluation of artificial intelligence identified ipratropium bromide for the treatment of coronavirus disease 2019
Source: Sci Rep. 2026 Feb 19;16:6980. doi: 10.1038/s41598-025-27869-y (PMC12920905; doi:10.1038/s41598-025-27869-y)
Supplement: Supplementary file 1 — Supplementary Material 1 [file 41598_2025_27869_MOESM1_ESM.docx]

**Supplementary Materials**

**Evaluation of artificial intelligence-identified ipratropium bromide for the treatment of coronavirus disease 2019**

The Supplementary Materials includes:

*1.* *Supplementary Methods*

*2. Supplementary Results*

*3. Supplementary Figures 1-3*

*4. Supplementary Table 1-3*

**Supplementary Methods**

**RAPTOR AI™**

The quality of results in the drug screening analyses relies on meticulous collection and preprocessing of datasets. After preparing RNA expression datasets, subsequent preprocessing, including outlier fitting, quantile normalization, and scale standardization, is performed to ensure consistency across batches (Figure 1a).

The DEG selection process uses the preprocessed RNA data, inputting all the transcript expression patterns into the algorithm. Subsequently, multiple statistical tests were employed to identify statistically significant gene expression differences between control and experimental samples (Figure 1b).

The drug-disease comparison identifies potential efficacy of a chemical in treating a target disease based on scores from contingency test (CT), enrichment test (ET), and similarity test (ST) using DEGs generated by both the disease and the chemical (Figure 1d). CT measures DEG contingency between drug and disease groups based on commonly expressed DEGs in both groups. ET calculates the relative distribution of the drug- or disease-related DEGs. ST identifies similarities in DEG expression patterns between drug and disease groups using vectorized DEG expression levels. A larger angle (θ) indicates lower similarity between drug and disease groups suggesting better suitability as the drug candidate for the target disease.

The score ensemble process ultimately calculates the final drug candidate score by summarizing three scores obtained from the drug-disease comparison. A higher score indicates hypothetically greater efficacy in modulating imbalanced gene expression patterns due to the target disease towards the normal state (Figure 1e).

All processed data will be archived for future studies and AI model development (Figure 1c).

**Hematoxylin and Eosin (H&E) staining**

Harris hematoxylin solution was used to stain the nuclei for 5 min for H&E staining. The slides were then washed with water, immersed in a 1% HCl-alcohol solution three times, and washed with running water. After bluing with a 1% ammonia solution, the cytoplasm was stained with an eosin solution for 3 min and immersed in 80%, 95%, and 100% ethanol three times for dehydration. The stained slides were dropped onto the mounting solution and covered with coverslips.

**Immunohistochemistry (IHC) staining**

For IHC staining, the slides were pretreated in a microwave oven for 15 min using 0.01 M citrate buffer (pH 6.0). Tissues were treated with 3% H_2_O_2_ for 10 min to inhibit peroxidase activity. Non-specific protein binding to antigens in tissues was blocked using normal serum. The Anti-Ly6g antibody (Neutrophil, Cat No. ab238132; Abcam, Cambridge, UK) incubated for 1 h and washed with a buffer solution. The secondary antibody was added for 30 min, followed by incubation with DAB (Cat No. K5007, Dako, Copenhagen, Denmark) for 5 min for color development. After washing with running water, Mayer's hematoxylin was counterstained for 1 min. The stained tissues were dehydrated for 5 min before mounting.

**Real time quantitative polymerase chain reaction (RT-qPCR)**

RT-qPCR was performed using a PrimerScript RT Reagent Kit (TaKaRa, Tokyo, Japan) according to the manufacturer's instructions. The forward and reverse primers used for RT-qPCR targeted the SARS-CoV-2 virus envelope (E) gene and were as follows: 5′-GCCTCTTCTCGTTCCTCATCAC-3′ and 5′-AGCAGCATCACCGCCATTG-3,’ respectively. The iQ SYBR Green SuperMix Kit (BioRad) was used for the RT-qPCR reaction under the following conditions: DNA denaturation at 95°C for 3 min and DNA amplification of 40 cycles of 15 s at 95°C and 30 s at 60°C.

**Supplementary Results**

**Inhibition of SARS-CoV-2 proliferation and inflammation in Vero cells without causing cytotoxicity by IB**

To evaluate the toxicity of remdesivir and ipratropium bromide in cells, a WST-1 assay was conducted to measure cell viability. The results showed that treatment with 4 μM of remdesivir and 0.001 and 0.01 μM of ipratropium bromide did not cause drug-induced cytotoxicity (Supplementary Table 1). Using non-toxic concentrations of remdesivir and ipratropium bromide, the SARS-CoV-2 virus copy number was measured using virus-specific primers. The results indicated a significant reduction in viral copy number in the remdesivir and ipratropium bromide treatment groups (Supplementary Figure 1).

To investigate the effect of ipratropium bromide on pro-inflammatory cytokines, we used an A549 cell line overexpressing ACE2-TMPRSS2, as SARS-CoV-2 binds to ACE2 and TMPRSS2 and increases ACE2 and TMPRSS2 upon infection. SARS-CoV-2 was introduced into A549-overexpressed ACE2-TMPRSS2 cells, and infection was confirmed by an increase in ACE2 and TMPRSS2 (Supplementary Figure 2a). The infected cells were co-cultured with THP-1 immune cells and treated with remdesivir and ipratropium bromide (Supplementary Figure 2b). The results demonstrated that the cytokines, such as interleukin (IL)-6 and IL-8, increased by the SARS-CoV-2 virus decreased in the remdesivir, ipratropium bromide-treated group (Supplementary Figure 2c).

**Supplementary figures**

**Supplementary Figure 1. Method for ipratropium bromide administered to SH101 hamsters
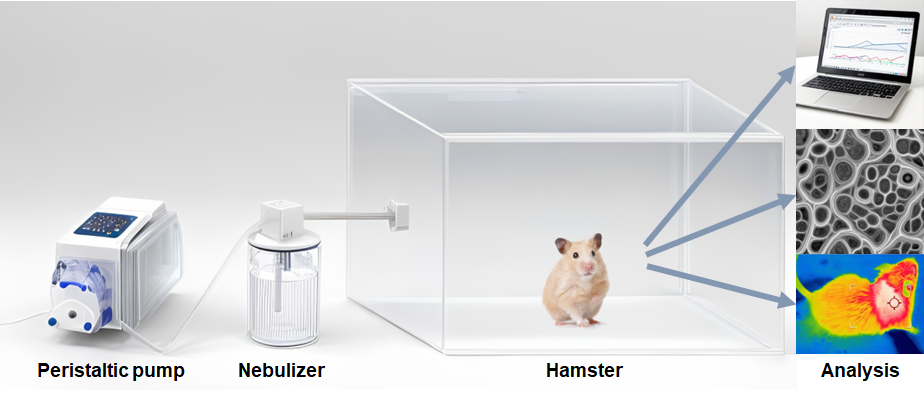
**

**Supplementary Figure 2. Evaluation of cell viability and antiviral activities of remdesivir (Rem) and ipratropium bromide (IB).** (a) Cell viability in Vero cells was measured using a WST-1 assay after treatment with remdesivir and ipratropium bromide for 48 h. (b) Vero cells were pre-infected with SARS-CoV-2 at 0.01 multiplicity of infection and treated with remdesivir and ipratropium bromide for 48 h. Quantitative polymerase chain reaction using SARS-CoV-2 virus-specific primers confirmed the virus copy number.

**
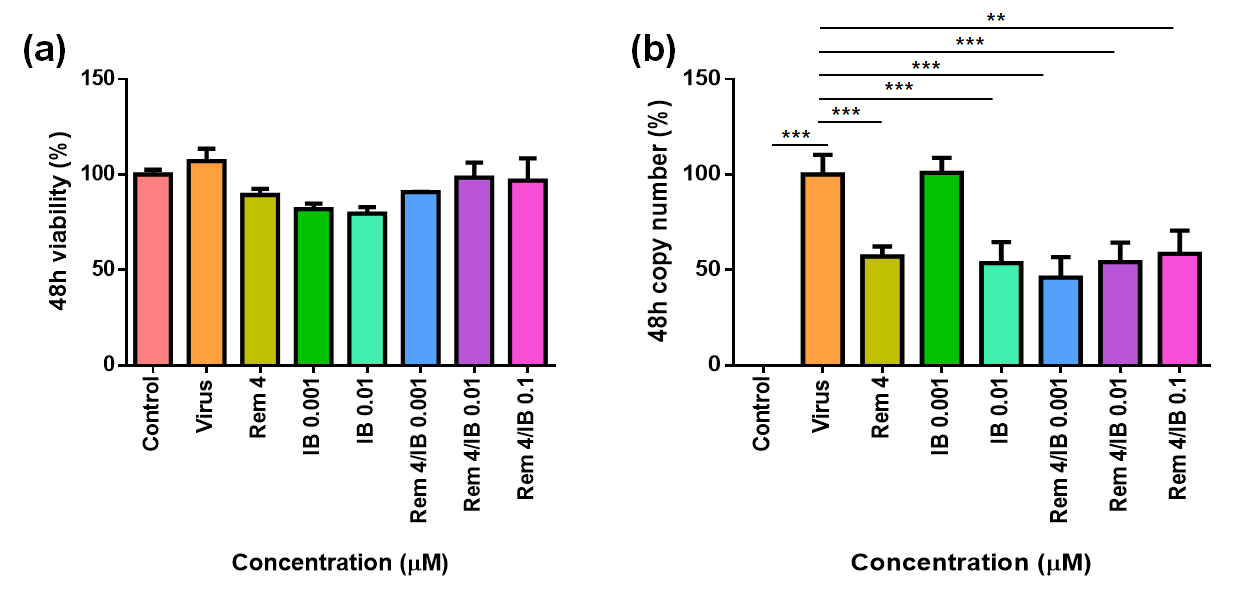
**

**Supplementary Figure 3. Changes of pro-inflammatory cytokines of remdesivir and ipratropium bromide on co-cultured and infected cells.** A: ACE2 (a) Verification of mRNA level changes of ACE2 and TMPRSS2 when co-cultured with SARS-CoV-2 virus-infected A549-ACE2-TMPRSS2 and THP-1 cells. (b) Cell morphology co-cultured with SARS-CoV-2 virus-infected A549-ACE2-TMPRSS2 and immune cell THP-1 cells. (c) Remdesivir and ipratropium bromide treatment for 48 h during co-culture with A549-ACE2-TMPRSS2 infected with SARS-CoV-2 virus and THP-1 cells, and changes in mRNA levels of interleukin (IL)-6 and IL-8 were confirmed. Statistical significance compared to the infection control group was indicated in the graph as * *P<*0.05, ** *P*<0.01, and *** *P<*0.001.


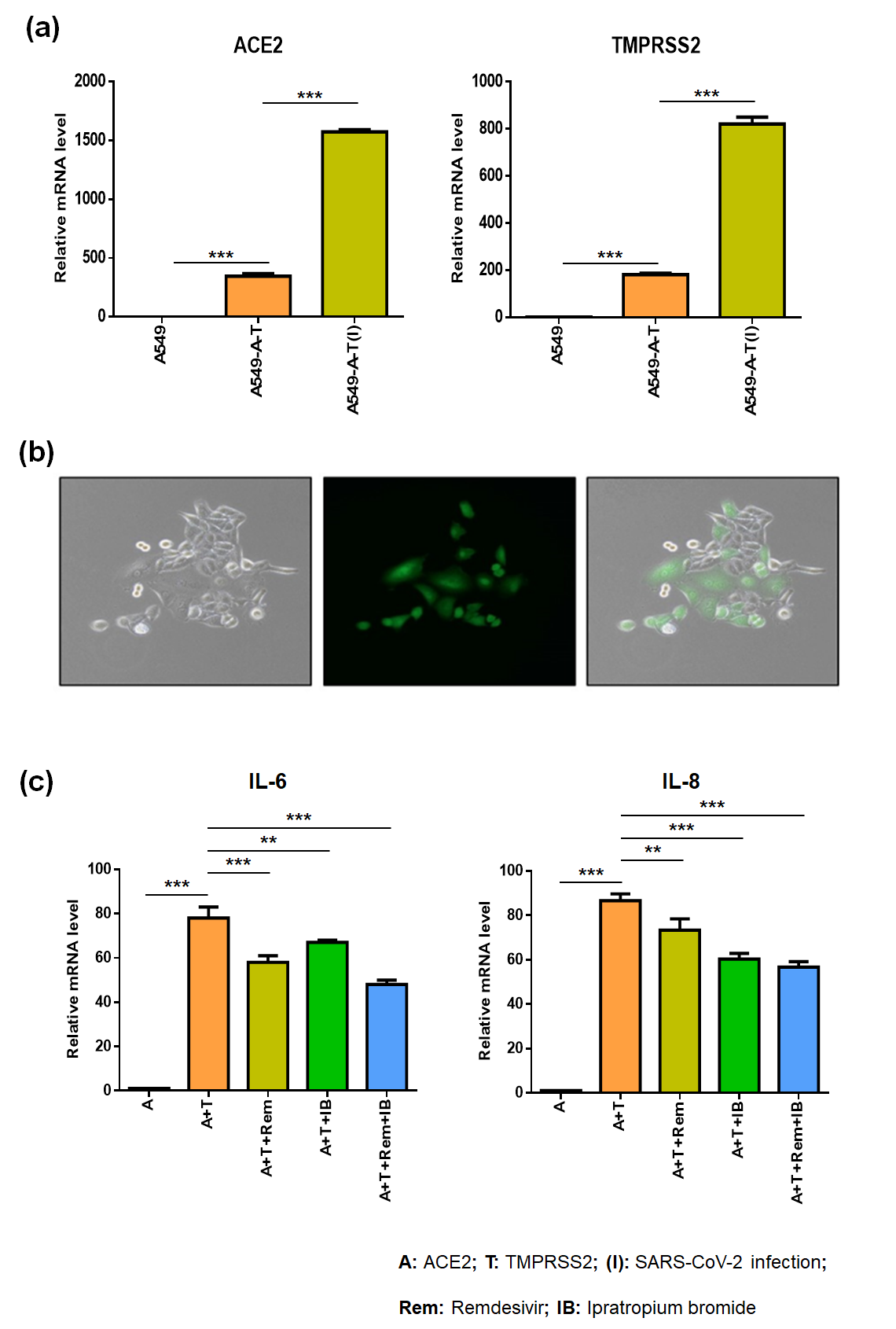


**Supplementary Tables**

**Supplementary Table 1. The differentially expressed genes used in the consensus pathway analysis (CPA)**

| Differently Expressed Genes (DEGs, 1% of total genes) |
| --- |
| RASL10B, NUP42, COL6A3, PRRG3, ZNF546, LRTOMT, CDHR4, CCDC36, PLA2G6, PPEF2, TCF7L1, SMARCAL1, PDHX, NSG1, IFT122, ZNF90, APOE, ZFP30, FLACC1, TKFC, PIDD1, CNPY4, RPL21P122, ZNF788P, AASS, CFTRP1, TBCCD1, CCDC127, TANGO6, CFHR1, STAP2, WNK4, TARS3, EBPL, C5, ZNF385A, JKAMP, NFS1, HMGN2P46, ECT2, KLK10, FAM122C, GPR173, LOC283922, DBR1, CCDC28A-AS1, TUBB4A, GREB1, ZNF567, ZNF799, KRT42P, ZNF726, CALCRL, SHOX, ITGB8, POU5F1, ZPR1, LOC101929583, RPS6P20, DNMT3A, PHC3, IGHVII-74-1, RNF2, DCTN5, HERC2P5, C6orf132, ZNF616, ZNF587, SUDS3, DHRSX, SPIN3, HSD17B13, CSTL1, FAM110D, LGMN, INMT, LSM12, HRG, GIMAP2, KBTBD12, ZNF251, IFT81, DUSP3, ZSCAN22, DLST, PNPO, SCN7A, CCDC122, SLC36A3, ECD, ZKSCAN3, SPACA6, RHBG, NRBP1, ZNF85, DCAF17, LDLRAD3, SHCBP1L, ABCA8, TBXA2R, TSPAN19, ATXN3, ZNF875, TTC23, CAMK2A, DIRAS2, COL28A1, TERB1, SF3B3, ZNF20, SNX27, PRXL2C, ANKRD40, RHD, PCDHB9, NMT2, TANGO2, AK7, POLR3K, GRIK2, KCTD8, SLC35F5, SPACA9, RNA5SP371, SYT5, FAM221A, GOLM1, DYDC1, KIF18B, PSMD10P2, GRAMD2A, SPTB, ZNF543, ADRA1A, LGI1, TRIM16L, LYRM4, OPHN1, ACTBP13, TPH1, CREG2, HLTF, ZNF221, RBM20, RAB12, GLMP, AAMDC, LIPC, HLA-V, PAICS, ZNF573, ZGRF1, LHFPL5, HNRNPKP5, RUVBL1, PSD3, REEP2, GYPA, B3GALT1, HMGB1P39, ZNF490, KIAA2012, CERS5, TBC1D30, CCDC86, RDH13, SMOC1, IFNLR1, TATDN3, MAPK9, NDRG2, SGTA, LRRC28, CTRC, SPATS2L, TVP23A, SLC15A1, MRPL10, ZNF544, SLC37A3, RNF227, MALL, LBHD1, ITSN1, MEFV, SLC16A4, PLA2G4C, ZNF347, FTLP6, PRR19, AKR1B1, CCDC200, XYLT2, PTCHD4, C17orf113, NIPSNAP1, ADGRV1, CYP2C9, CYBRD1, CFAP97D1, DNAJC14, RSPH10B, GAL3ST4, PAEP, MAGI2, C10orf82, IFI27, COL8A1, LIPT1, CLCN2, DPPA4, TINF2, FBXO44, LEPROTL1, EXOSC2, SMPD3, PHYHD1, INKA2, PSPH, CATSPERG, GSDMA, SLC48A1, NUP43, CCDC158, CABYR, SHC1, TCEANC2, SIRT5, TMEM150C, TLCD3B, DAND5, SNX11, AGAP4, CYP4V2, ZNF141, TERF2, H2AC11, SDK2, TRMT12, OR9K1P, COX6B2, HOGA1, ZNF2, EHD2, C12orf43, STRIP2, CD151, FAIM, FAM83H-AS1, GEMIN2, MAP1LC3C, RBFA, SLC27A5, MRPS17, EHMT2, RBMS2, NIM1K, C1orf174, ACBD4, SLC37A2, RASSF6, PRICKLE2, FARP1, ZZZ3, MTND4P30, TERB2, ADSS1, TM4SF19, ZFP2, STEAP2, MS4A10, ULK2, ACADL, YES1, SLC2A5, KAT8, PLS1, CLVS1, HACD2, CANX |

**Supplementary Table 2. Study design of the evaluation of COVID-19 therapeutic efficacy**

| **Group** | **SARS-CoV-2**  **(10^5^ TCID_50_)** | **Viral infection &**  **drug administration** | **Administration route** | **Time of drug administration (days post-infection)** |
| --- | --- | --- | --- | --- |
| G1; Control | - | Saline 100 μL | Intranasal | 1–8 |
| G2; Vehicle | + | Saline 100 μL | Intranasal | 1–8 |
| G3; Positive control | + | Remdesivir  15 mg/kg (100 μL) | Intravenous | 1–8 |
| G4; Test article | + | Ipratropium bromide  62.5 μg/kg (2 mL) | Inhalation (2 h) | 1–8 |

**Supplementary Table 3. Eight representative immune and inflammatory response-related pathways were highly ranked in the consensus pathway analysis** (**CPA) pathway analysis**

| **Pathway Name** | **No. of Genes Enriched** | **Wilcox-FDR** |
| --- | --- | --- |
| Natural killer cell-mediated cytotoxicity | 131 | 1.11E-07 |
| Autophagy – animal | 137 | 0.000164639 |
| Th17 cell differentiation | 107 | 4.16E-12 |
| T cell receptor signaling pathway | 104 | 3.90E-08 |
| B cell receptor signaling pathway | 82 | 2.36E-08 |
| PD-L1 expression and PD-1 checkpoint pathway in cancer | 89 | 5.42E-08 |
| Primary immunodeficiency | 38 | 1.14E-07 |
| Th1 and Th2 cell differentiation | 92 | 6.38E-12 |
